# Prevalence and factors associated with postpartum depressive symptoms among mothers who gave birth within the past 12 months in Ghana: mixed-method study

**DOI:** 10.1192/bjo.2025.10857

**Published:** 2025-10-14

**Authors:** Leticia Tornyevah, Samuel Bosomprah, Anjali Sharma, Ank De Jonge, Jens Henrichs

**Affiliations:** Midwifery Science, Location Vrije Universiteit Amsterdam, Amsterdam UMC, Amsterdam, The Netherlands; Midwifery Academy Amsterdam Groningen, InHolland, Amsterdam, The Netherlands; Mental Health, Amsterdam Public Health, Amsterdam, The Netherlands; Department of Primary and Long Term Care, University Medical Center Groningen, University of Groningen, Groningen, The Netherlands; Research Department, Centre for Infectious Disease Research in Zambia, Lusaka, Zambia; Department of Biostatistics, School of Public Health, University of Ghana, Accra, Ghana; Amsterdam Reproduction & Development Institute, Amsterdam, The Netherlands

**Keywords:** Postpartum depressive symptoms, mothers, prevalence, associated factors, pathways

## Abstract

**Background:**

Postpartum depressive symptoms (PPDS) are mental health concerns, characterised by sadness, anxiety and suicidal ideation.

**Aims:**

We aimed to estimate the prevalence of PPDS, identify its associated factors and explore the lived experiences of individuals with PPDS, to understand the psychosocial mechanisms involved.

**Method:**

We surveyed 400 women aged 18 years and above and conducted in-depth interviews among 19 women who screened positive for PPDS at two urban hospitals and one peri-urban polyclinic in Ho, Ghana. We used multivariable binomial generalised linear models to identify factors independently associated with PPDS. We used thematic analysis (qualitative) to identify themes that highlight pathways through which these factors influence PPDS.

**Results:**

Overall, 117 (29.3%) women screened positive for PPDS. Being unmarried (adjusted prevalence ratio (aPR) 1.33, 95% CI 1.02–1.72), lack of partner support (aPR 1.60, 95% CI 1.21–2.12), history of depressive or psychiatric disorders (aPR 2.44, 95% CI 1.84–3.25), unplanned pregnancy (aPR 1.63, 95% CI 1.18–2.25), low self-esteem (aPR 2.38, 95% CI 1.79–3.16) and low birth weight (aPR 1.87, 95% CI 1.33–2.65) were independently associated with PPDS. The thematic analysis revealed four key themes: (a) social isolation and limited support, (b) emotional stress and vulnerability, (c) self-image and identity challenges, and (d) resilience resources.

**Conclusions:**

PPDS were common in our sample and were significantly associated with modifiable factors such as prior mental health history and low self-esteem. These findings underscore the importance of prioritising maternal mental health through the integration of depression screening and psychosocial care into routine antenatal and postnatal services.

Postpartum depressive symptoms (PPDS) are a significant but often overlooked mental health problem. Globally, about 10–25% of women experience significant depressive symptoms after childbirth.^1^ Sub-Saharan Africa appears to bear a particularly high burden, with a pooled prevalence around 22%;^1^ reported rates vary widely.^1^ In Ghana, studies likewise report highly variable rates of PPDS; community-based surveys in northern Ghana often find rates in the mid-20% range, whereas urban facility-based studies (e.g. in Greater Accra) tend to find much lower rates.^2^ Overall, reported prevalences of PPDS in Ghana have ranged from the single digits up to about 40%, with pooled estimates on the order of only 9–10%.^2^ These disparities may suggest that many cases go unrecognised. Cultural stigma and low awareness contribute to underdiagnosis; nearly half of Ghanaian mothers with PPDS may remain untreated because of stigma and privacy concerns.^2^ Mental health services are scarce and unevenly distributed in Ghana; all three large psychiatric hospitals are located on the southern coast,^3^ leaving rural and northern areas particularly underserved. Moreover, just recently, the Ghanaian Government started to invest more into mental healthcare services, and until the end of 2024, healthcare costs related to the treatment of major mental health problems, like depression or bipolar disorder, were not financially covered by health insurance in Ghana.^4^

Identifying the factors associated with PPDS and understanding how these factors contribute to PPDS are critical for optimising culturally sensitive screening tools, intervention strategies and support programmes to improve maternal and child health. Several factors, such as a lack of spousal support,^5^ low self-esteem,^6^ unplanned pregnancies^5^ and low birth weight,^7^ are known to increase the risk of developing PPDS. These factors often work together with social and psychological dynamics, placing significant emotional strain on mothers during the postpartum period. For example, women without a supportive partner often feel isolated and overwhelmed, harbouring persistent negative self-appraisal.^8,9^ Unplanned pregnancies can create additional financial and emotional pressures.^8^ Caring for a low-birth-weight baby may lead to increased anxiety and self-doubt about parenting abilities,^7^ further increasing their vulnerability to depressive symptoms.^10,11^ Most of the existing research in Ghana, however, has relied on surveys,^2^ with little exploration of the underlying mechanisms that can explain the quantitative data; for example, how mothers personally experience and cope with PPDS in their daily lives. There is a clear paucity of qualitative data on the lived experiences, social dynamics and cultural context underlying PPDS among Ghanaian women. This evidentiary gap in understanding why and how PPDS unfolds in the Ghanaian context suggests the need for a more holistic investigative approach. A mixed-methods study design is well suited to address this gap.

The general aim of this study was to provide a better understanding of PPDS among mothers in Ghana. Specifically, we sought to estimate the prevalence of PPDS among mothers within a year postpartum; identify sociodemographic, psychosocial and maternal factors independently associated with its onset; and explore lived experiences of affected mothers to better understand the emotional, psychological and social challenges contributing to PPDS.

## Method

### Study design and settings

We conducted an explanatory sequential, mixed-methods, cross-sectional study, beginning with a cross-sectional survey followed by in-depth interviews, at the Ho Teaching Hospital, Ho Municipal Hospital and Ho Polyclinic, from September 2024 to November 2024. We selected the Ho Teaching Hospital and Ho Municipal Hospital (larger urban hospitals) and Ho Polyclinic (serving peri-urban/rural outskirts) because together they serve most births in the Ho area. Ho is the Volta regional capital situated in South Ghana. The design allows quantitative findings to guide qualitative exploration of underlying mechanisms.^12^

### Participants and sampling

Eligible women were those aged 18 years and above, who delivered live infants between September 2023 and September 2024 (i.e. those with a child younger than 12 months). We recruited mothers as they attended postnatal or immunisation clinics at the three facilities. Research assistants informed all eligible women present during data collection days about the study and invited them to participate; those unable or unwilling to consent were excluded. We approached 405 mothers, of which five were excluded (one mother was below 18 years of age, and four mothers had a child older than 12 months), giving an eligibility rate of 98.8%. Of the 400 eligible mothers surveyed, one mother had missing data, leaving 399 (99.8% of those eligible) for analysis.

### Sample size considerations

The sample size was calculated based on an assumed prevalence of PPDS in Ghana of 20.3%.^2^ We used a single-group design to obtain a two-sided 95% confidence interval for a single proportion. To produce a confidence interval with a width of no more than 0.1, at least 246 mothers were needed. The sample size was computed using PASS 2023, version 23.0.2.

We planned purposive sampling of mothers with elevated depressive symptoms. A sample size of 19 in-depth interviews was deemed adequate, as qualitative literature indicates thematic saturation is often reached with 12–15 interviews.^13^ Indeed, our coding process indicated no new themes emerging in the last few interviews.

### Data collection

Following consent, trained research assistants administered the structured questionnaire using Android tablets, in the participant’s preferred language (Ewe or English). All survey data were collected using Castor EDC version 2024.3.4.0 (Castor, Philadelphia, PA, USA; see https://www.castoredc.com/), a certified electronic data capture tool hosted at a secure server of the Amsterdam University Medical Center, for macOS. We incorporated validation checks in the digital survey to optimise data quality assurance and control. The use of anonymous tablets for data collection helped minimise social desirability. We pilot-tested the questionnaire on a convenience sample of ten postpartum women at a nearby clinic and refined wording as needed.

#### Interview procedures and setting (Consolidated Criteria for Reporting Qualitative Research (COREQ): items 10–15, 17–23)

Interviews were conducted face-to-face in private space within the child welfare clinics at the three facilities, with only the participant and interviewer present; infants were present when breastfeeding was required (no other non-participants were present). After each survey, women with a Patient Health Questionnaire-9 (PHQ-9) score >9 (indicative of PPDS), as automatically identified by the electronic data capture tool, were asked about interest in a follow-up in-depth interview by the trained research assistants. The research assistant took contact and location details from interested women. Women signed an informed consent form before participating in the interview. We encouraged participants eligible for the interview to see a clinician or a nurse to discuss PPDS symptoms and to return to the clinic the next day for an in-depth interview (no repeat interviews). Participants were informed that their care would not be affected by their responses.

A semi-structured interview guide (developed from the literature and the study objectives) covered topics such as experiences during childbirth, postpartum emotional well-being, support from family and community, stressors and coping strategies (Supplementary Table 1 available at https://doi.org/10.1192/bjo.2025.10857). The guide was pilot-tested with two postpartum women from a non-study clinic to refine wording and flow. The average duration of the interview was 45 min. We recorded, with permission, all interviews with a Sony ICD digital voice audio recorder. Brief field notes were written immediately after each interview to capture contextual details and initial analytic impressions. We discussed data sufficiency at regular intervals and concluded that thematic saturation had been reached when no new themes emerged in the final interviews. Transcripts were not returned to participants for correction because of time constraints and to minimise participant burden. Instead, we prioritised clear in-interview summaries and probes to enhance credibility.

We gave GHC45 (approximately US$3) to all survey and interview participants as appreciation for their time. Their familiarity with the culture helped elicit the most accurate responses.^14^

#### Research team and reflexivity (COREQ: items 1–8)

Interviews were conducted by L.T. (female, PhD candidate in Mental Health), supported by two female research assistants fluent in Ewe and English. The core team comprised L.T. (PhD candidate), S.B. (PhD, biostatistics), A.S. (ScD, social science), A.D.J. (PhD, midwifery science) and J.H. (PhD, perinatal mental health). At the time of the study, team members were employed at academic or research institutions in Ghana, The Netherlands and Zambia. Interviewers received study-specific training on trauma-informed interviewing, confidentiality and reflexive practice. They conducted mock interviews and reviewed role-plays before data collection. No prior relationships existed with participants. During recruitment, the interviewer introduced herself as a researcher studying postpartum well-being, emphasising the voluntary nature of participation and that there were no right or wrong answers. We maintained a reflexive log to document positionality, assumptions and analytic decisions. The team’s clinical and public health backgrounds were acknowledged as potential sources of interpretive bias. To mitigate these, we used peer debriefs and multidisciplinary review of codes and themes.

#### Outcome and covariates

The PHQ-9 was used to measure our primary outcome, defined as screening positive for PPDS. The PHQ-9 is a nine-item scale with responses scored on a four-point scale (0 = not at all; 1 = several days; 2 = more than half the days; 3 = nearly every day). The total score ranges from 0 to 27. We used a PHQ-9 score >9 as the cut-off to indicate elevated depressive symptoms, based on evidence indicating this threshold provides strong predictive validity.^15^ Although the Edinburgh Postnatal Depression Scale (EPDS) is a widely recognised tool specifically designed for PPDS, we selected the PHQ-9 scale because of its broader applicability, strong correlation with the EPDS and demonstrated reliability and validity for detecting PPDS, with a sensitivity of 0.84 and specificity of 0.81.^16^ Similarly, the PHQ-9 has been validated in Ghana (e.g. Kintampo district) with satisfactory reliability (Cronbach’s alpha of approximately 0.75) and concordant performance with the EPDS.^17^ Importantly, the PHQ-9 offers practical advantages in this context, being simple, easy to administer and well-suited to Ghanaian women, particularly those with limited formal education, where its straightforward format and accessibility enhance its acceptability and feasibility.^17^

We collected sociodemographic data (e.g. age, education, marital status, employment, etc.), pregnancy and obstetric history (e.g. parity, pregnancy planning), and psychosocial factors (e.g. partner support, stressful life events, self-esteem) that are potentially related to PPDS based on the literature.^5,10^ We defined ‘in union’ as being married or cohabiting, and ‘not in union’ as women who were single, divorced, separated or widowed. Cohabiting women were grouped with married women because of sociocultural similarity in support structures in Ghana. We asked mothers a structured question, ‘Has your husband/partner been supportive since delivery? (e.g. providing emotional, financial or practical help)’. We defined ‘partner support’ as ‘supportive’ if moderate/strong support was reported, and ‘unsupportive’ if the mother reported no or poor support from husband/partner. Pregnancy was classified as planned if the mother reported intending the pregnancy; otherwise, it was ‘unplanned’. Low birth weight was defined as <2.5 kg. We asked women if they had any paid work (employment or self-employment) at the time of participating in the survey. Those answering ‘yes’ to any paid work were coded as ‘employed or engaged in paid work’; others (including housewives without income) were coded as ‘unemployed or not working’.

We used the Childbirth Experience Questionnaire 2 (CEQ2) to measure mothers’ experiences during childbirth and labour. The CEQ2 consists of 22 items. For 19 items, we used a four-point Likert scale (1 = totally agree, 2 = mostly agree, 3 = mostly disagree, 4 = totally disagree). Higher scores indicate a negative experience. We assessed three additional items (i.e. labour pain, sense of security and control) using a visual analogue scale (VAS). We categorised VAS scores as follows: 0–40 = 1, 41–60 = 2, 61–80 = 3 and 81–100 = 4, with higher scores indicating a more positive experience.^18,19^ We reversed items with increasing scores, indicating positive experience before calculating the total score, summing all individual four-point items, and expressed as a percentage. All 22 items (19 Likert items, three converted VAS items) were summed for a total score (minimum 22, maximum 88) and expressed as a percentage. Based on the CEQ2 manual and aligning with the four-point Likert scale, having a median of 50%, we classified CEQ2 scores >50% as negative childbirth experience and ≤50% as positive childbirth experience.^18,19^

We used the Stressful Life Event Screening Questionnaire to assess stressful life events experienced by mothers throughout their lives. This 13-item questionnaire includes binary (yes/no) responses, and a higher scores indicate greater exposure to stressful life events.^20^ Moderate-to-high stress was defined as scores ≥4. To measure self-esteem, we used the Rosenberg Self-Esteem Scale (RSE), a ten-item scale with four response options (strongly agree, agree, strongly disagree, disagree). We reversed negative items and calculated the total score by summing all individual four-point items, with higher scores indicating higher self-esteem.^21^ We defined low self-esteem as scores ≤14.^22^ We pre-tested the instruments for comprehension; however, formal validation of the CEQ2 in Ghana was not performed.

### Data analysis

#### Quantitative data analysis

We summarised background characteristics of mothers by using frequency and proportions for categorical variables, whereas median and interquartile interval (IQI) were used for continuous variables. We used the Pearson chi-squared test or Fisher’s exact test, as appropriate, to assess the association of potential factors associated with PPDS. To develop a parsimonious model of factors independently associated with PPDS, variables with a *P*-value less than or equal to 0.05 were considered as candidates for inclusion in the multivariable binomial generalised linear model (GLM). Variables were removed if *P*-values were larger than 0.1. We chose the binomial GLM with a log-link to estimate the prevalence ratio because the odds ratio would overestimate the effect owing to the high prevalence of PPDS in our sample. Missing responses were rare (i.e. one item-nonresponse) because of electronic validation checks; analyses were performed on complete cases. All analyses were performed using Stata 18 MP for macOS.

#### Qualitative analysis and trustworthiness (COREQ: items 24–28; reporting 29–32)

We used reflexive thematic analysis following Braun and Clarke’s six steps: (1) familiarisation with data, (2) generation of initial codes, (3) searching for themes, (4) reviewing themes, (5) defining and naming themes and (6) writing the report.^23^ Interviews (*n* = 2) were recorded in the local language (Ewe) and translated into English. All interviews were transcribed verbatim, and detailed notes were compiled, ensuring that all responses were labelled with unique participant identifiers. After familiarisation with the interview data, transcripts and notes, L.T. developed a coding framework. L.T. coded the data systematically, tagging relevant sections with descriptive codes, combining inductive development of data-driven codes with deductive attention to constructs identified *a priori* from the literature and the quantitative findings. These codes were then grouped into broader themes. A.S. independently reviewed a subset of the transcripts, and discrepancies were discussed with A.D.J., J.H. and S.B. until interpretive consensus was reached. Finally, L.T. synthesised the results into a coherent narrative, highlighting key themes illustrated by representative maternal quotes. We managed data and coding using Dedoose version 9.0 for macOS (Sociocultural Research Consultants, LLC, Los Angeles, California, USA; see https://www.dedoose.com).

We provided an excerpt of the coding tree with definitions and example quotations in Supplementary Table 2. We assessed credibility through integration with the quantitative results. We compared statistically significant factors with emergent qualitative themes, via a joint display and narrative, to interpret how these findings corroborate or extend one another.^12^ Although member-checking of full findings with participants was not undertaken to reduce burden, we incorporated in-interview summaries and clarification probes, and we present diverse and negative cases alongside the major themes to support dependability and confirmability. We followed the Strengthening the Reporting of Observational Studies in Epidemiology (STROBE) guidelines (Supplementary Table 3) in reporting the cross-sectional component,^24^ whereas the qualitative findings followed the COREQ checklist^25^ (Supplementary Table 4).

## Results

### Background characteristics of mothers

We approached 405 mothers, of whom five were excluded (one mother was under 18 years of age, and four mothers had a child younger than 12 months), giving an eligibility rate of 98.8%. Of the 400 mothers who participated in the survey, one had no survey data, leaving a total of 399 (99.8%) for analysis. For these, the median age was 30 years (IQI: 26.0–34.0). Two-thirds (*n* = 260, 65.0%) had at least a secondary education, 323 (80.8%) were engaged in paid work (including self-employment) and 343 (86.0%) were married (Table 1). Furthermore, 120 (30.1%) had a history of depressive or psychiatric disorder (Table 2). In total, 75 (18.8%) women had a baby with complications at birth (Table 3).

**Table 1:** Sociodemographic and social characteristics of mothers who gave birth within the past 12 months

Characteristics	Number of participants (% of total)
*N*	400
Age (years), median (IQI)	30 [26–34]
Age group (years)	
18–24	68 (17.0)
25–34	259 (64.8)
≥35	73 (18.2)
Education	
None/primary	140 (35.0)
Secondary or higher	260 (65.0)
Occupation	
Employed (formal/self)	323 (80.8)
Unemployed	77 (19.2)
Location	
Urban	380 (95.2)
Rural	19 (4.8)
Marital status	
In union	344 (86.0)
Not in union	56 (14.0)
Religion	
Christian	378 (94.5)
Other	22 (5.5)
Partner support	
Supportive	353 (88.5)
Unsupportive	46 (11.5)
History of depressive/ other psychiatric disorder	
No	279 (69.9)
Yes	120 (30.1)
Family history of psychiatric illness	
No	381 (95.5)
Yes	18 (4.5)

IQI, interquartile interval.

**Table 2:** Maternal characteristics of mothers who gave birth within the past 12 months

Characteristics	Number of participants (% of total)
Current pregnancy planned	
Yes	227 (56.9)
No	172 (43.1)
Type of pregnancy	
Singlet pregnancy	381 (95.5)
Multiple pregnancy	18 (4.5)
Parity	
Primiparity	164 (41.1)
Multiparity	235 (58.9)
Body mass index (BMI)	
Obese	116 (29.1)
Overweight	126 (31.6)
Healthy weight	147 (36.8)
Underweight	10 (2.5)
Self-esteem (RSE)	
Normal/high self-esteem	372 (93.2)
Low self-esteem	27 (6.8)
Childbirth experience (CEQ2)	
Positive childbirth experience	90 (22.6)
Negative childbirth experience	309 (77.4)
Stressful life event	
Low stress exposure	368 (92.2)
Moderate/high stress exposure	31 (7.8)
Obstetric complications	
No	231 (57.8)
Yes	169 (42.2)
History of medical condition	
No	372 (93.0)
Yes	28 (7.0)

RSE, Rosenberg Self-Esteem Scale; CEQ2, Childbirth Experience Questionnaire version 2.

**Table 3:** Characteristics of babies born to mothers who gave birth within the past 12 months

Characteristics	Number of participants (% of total)
Gender of the baby	
Male	206 (51.6)
Female	193 (48.4)
Baby had complications at birth	
No	325 (81.2)
Yes	75 (18.8)
Gestation	
Term	363 (91.0)
Preterm	36 (9.0)
Baby admitted to hospital within the past 6 weeks	
No	378 (94.7)
Yes	21 (5.3)
Baby has difficulty in breastfeeding	
No	383 (96.0)
Yes	16 (4.0)
Baby has difficulty in sleeping	
No	376 (94.2)
Yes	23 (5.8)
Birth weight (medium/IQI)	3 [3–4]
Birth weight	
Normal	356 (89.2)
Low	43 (10.8)

IQI, interquartile interval.

### Prevalence and factors associated with PPDS

**Fig. 1:**
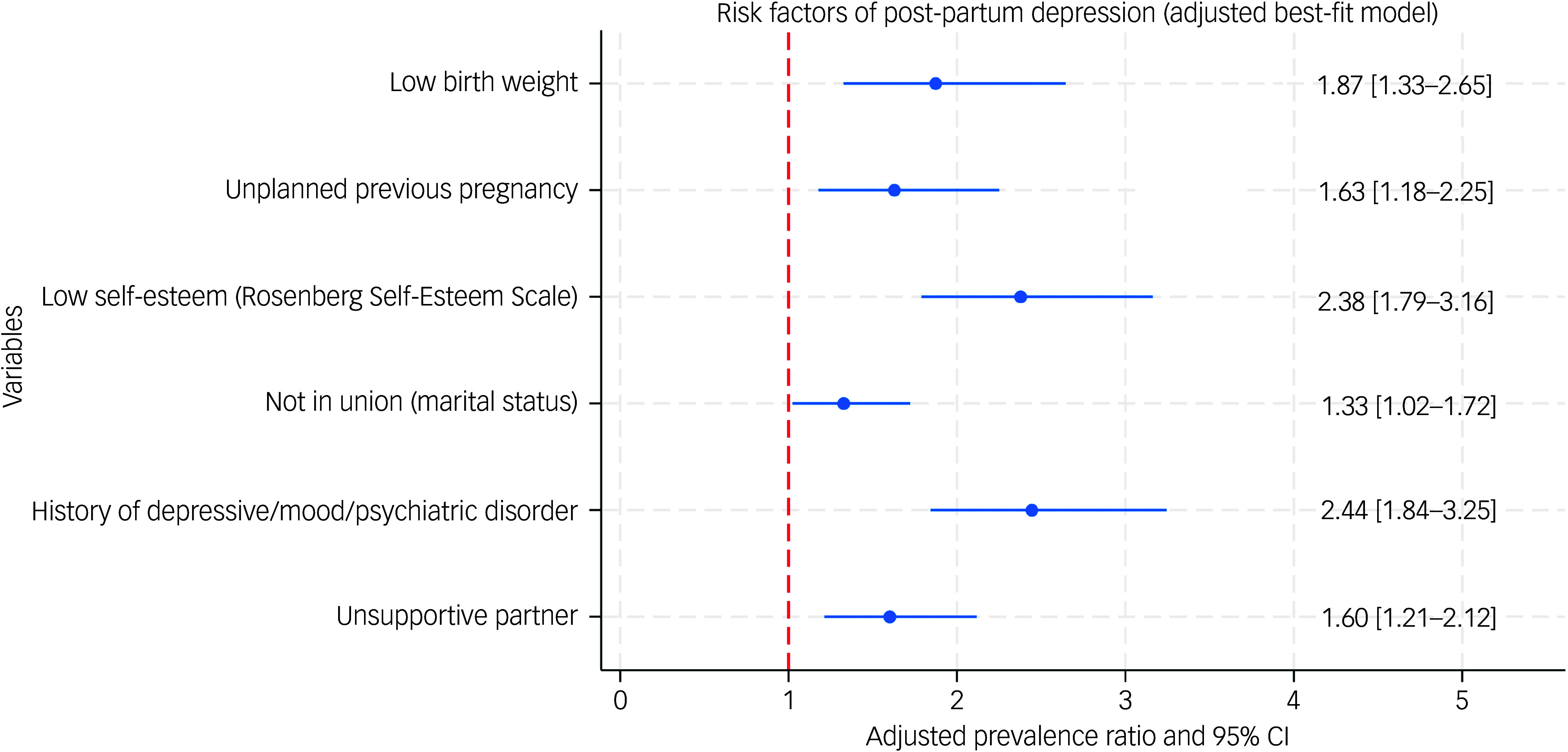
Forest plot of factors independently associated with postpartum depression.

Of 399 mothers, 117 (29.3%) screened positive for PPDS (Table 4). The multivariable binary regression results showed that several demographics, psychosocial and perinatal factors were independently associated with PPDS (Fig. 1, Tables 4 and 5). Being unmarried was associated with an increased risk of developing PPDS (adjusted prevalence ratio (aPR) 1.33, 95% CI 1.02–1.72; *P* = 0.034). Additionally, lack of partner support was linked with a higher likelihood of developing PPDS (aPR = 1.60, 95% CI 1.21–2.12; *P* = 0.001) (Table 4). A history of depressive or psychiatric disorder was also associated with PPDS (aPR = 2.44, 95% CI 1.84–3.25; *P* < 0.001) (Table 4). Furthermore, unplanned pregnancy (aPR = 1.63, 95% CI 1.18–2.25; *P* = 0.003) and low self-esteem was associated with an increased risk of PPDS (aPR = 2.38, 95% CI 1.79–3.16; *P* < 0.001) (Table 5). Finally, low birth weight was also associated with a higher risk of PPD (aPR = 1.87, 95% CI 1.33–2.65; *P* < 0.001) (Table 5).

**Table 4:** Sociodemographic and social factors of postpartum depression among mothers who gave birth within the past 12 months

Associated factors	Number (%) screened positive for postpartum depression	Univariable model		Multivariable model	
		Prevalence ratio [95% CI]	*P*-value	Prevalence ratio [95% CI]	*P*-value
Overall prevalence [95% CI]	117 (29.3)	[25.1–34.0]			
Age group (years)					
18–24	28 (41.2)	Reference			
25–34	71 (27.5)	0.67 [0.47–0.95]	0.023		
≥35	18 (24.7)	0.60 [0.37–0.98]	0.041		
Education					
None/primary	51 (36.7)	Reference			
Secondary or higher	66 (25.4)	0.69 [0.51–0.94]	0.017		
Occupation					
Employed (formal/self)	88 (27.3)	Reference			
Unemployed	29 (37.7)	1.38 [0.98–1.93]	0.063		
Location					
Urban	107 (28.2)	Reference			
Rural	10 (52.6)	1.87 [1.18–2.95]	0.007		
Marital status					
In union	90 (26.2)	Reference		Reference	
Not in union	27 (48.2)	1.84 [1.33–2.54]	<0.001	1.33 [1.02–1.72]	0.034
Partner support					
Supportive	85 (24.1)	Reference		Reference	
Unsupportive	32 (69.6)	2.89 [2.21–3.77]	<0.001	1.60 [1.21–2.12]	0.001
History of depressive or other psychiatric disorder					
No	51 (18.3)	Reference		Reference	
Yes	66 (55.0)	3.01 [2.24–4.05]	<0.001	2.44 [1.84–3.25]	<0.001

**Table 5:** Maternal and paediatric factors of postpartum depression among mothers who gave birth within the last 12 months

Associated factors	Number (%) screened positive for postpartum depression	Univariable model		Multivariable model	
		Prevalence ratio [95% CI]	*P*-value	Prevalence ratio [95% CI]	*P*-value
Current pregnancy planned					
Yes	41 (18.1)	Reference		Reference	
No	76 (44.2)	2.45 [1.77–3.38]	<0.001	1.63 [1.18–2.25]	0.003
Type of pregnancy					
Singlet pregnancy	108 (28.4)	Reference			
Multiple pregnancy	9 (50.0)	1.76 [1.08–2.88]	0.023		
Self-esteem (RSE)					
Normal/high self-esteem	93 (25.0)	Reference		Reference	
Low self-esteem	24 (88.9)	3.56 [2.85–4.44]	<0.001	2.38 [1.79–3.16]	<0.001
Childbirth experience (CEQ2)					
Positive childbirth experience	79 (24.8)	Reference			
Negative childbirth experience	38 (46.9)	1.89 [1.40–2.55]	<0.001		
Stressful life event					
Low stress exposure	102 (27.7)	Reference			
Moderate/high stress exposure	15 (48.4)	1.75 [1.17–2.60]	0.006		
Obstetric complications					
No	56 (24.4)	Reference			
Yes	61 (36.1)	1.48 [1.09–2.01]	0.011		
Baby had complications at birth					
No	87 (26.9)	Reference			
Yes	30 (40.0)	1.49 [1.07–2.07]	0.018		
Birth weight					
Normal	97 (27.3)	Reference		Reference	
Low	20 (46.5)	1.71 [1.19–2.45]	0.004	1.87 [1.33–2.65]	<0.001

RSE, Rosenberg Self-Esteem Scale; CEQ2, Childbirth Experience Questionnaire version 2.

### Qualitative findings

We conducted 19 in-depth interviews with mothers experiencing depressive symptoms to explore how various factors contribute to PPDS within the context of their lived experiences. The background characteristics of interview participants is presented in Supplementary Table 5. Our analysis identified four key themes that highlight the interconnected social, psychological and emotional pathways through which these factors influence PPDS. The themes included (a) social isolation and limited support, (b) emotional stress and vulnerability, (c) self-image and identity challenges, and (d) resilience resources.

#### Social isolation and limited support

Women frequently described experiencing profound social isolation because of a lack of physical, emotional and financial support from their partners. Some mothers shared feelings of abandonment during critical moments such as labour, delivery and postpartum recovery, as their husbands were often absent. One mother remarked, ‘He wasn’t there when I needed him most, especially during labour and those first few weeks after birth’ (P01, 34 years, married). Emotional unavailability from partners further compounded feelings of loneliness, as mothers struggled to deal with the challenges of motherhood without a confidant. As one mother reflected, ‘I needed someone to talk to, someone to understand how I feel, but my partner just wasn’t there emotionally’ (P02, 32 years, cohabiting). This lack of emotional support often left mothers overwhelmed, showing their sense of helplessness during recovery and newborn care.

Financial strain also contributed to feelings of isolation and vulnerability, particularly for unmarried mothers. One unmarried mother shared, ‘I’m unmarried, and my partner doesn’t provide enough. I feel like I’m carrying everything on my own’ (P11, 27 years, not in union), and another, who was breastfeeding twins, lamented, ‘My partner barely supports us financially. I feel overwhelmed and alone’ (P03, 42 years, married). These challenges often left mothers without the essential support they needed, increasing their susceptibility to mental and emotional distress.

For unmarried mothers, the isolation was exacerbated by societal criticism of being unmarried before giving birth. Many spoke of judgement, shame, rejection and stigma because of social expectations of marriage before motherhood and experiences of rejection by their own family. One mother revealed, ’Society expects us to marry first, then have children. Because I didn’t, people insult me. It hurts me every day’ (P18, 22 years, not in union), and another shared, ‘My family said I’m a disgrace for giving birth without being married. It makes me feel so alone and ashamed. (P11, 27 years, not in union).

Some women expressed being confronted with family disappointment, which deepened their feelings of failure, as one mother explained, ‘My family said I disappointed them because I got pregnant before finishing school. They made me feel like a failure’ (P19, 20 years, not in union). Younger mothers also experienced social exclusion, often feeling judged and alienated by their peers. One younger mother explained, ‘When I got pregnant at 19, my friends started avoiding me. They insulted me, and I felt ashamed’ (P05, 19 years, cohabiting). This distancing by friends intensified feelings of loneliness and social exclusion.

#### Emotional distress and vulnerability

Mothers described emotional distress, often exacerbated by the lack of support, the demands of caregiving and the physical recovery process. These challenges were particularly pronounced among mothers with a history of mental health conditions. Many mothers recounted, ‘I’ve had depression before, but this time it feels worse because my family has rejected me, and I’m doing everything alone’ (P05, 19 years, cohabiting). Moreover, many mothers described how pre-existing mental health challenges resurfaced during the postpartum period, intensified by the emotional and physical demands of motherhood. One mother reflected, ‘I was already struggling mentally before I got pregnant. After the baby came, it became worse because I had no one to talk to’ (P06, 31 years, separated).

#### Self-image and identity challenges

Mothers frequently described emotional strain linked to feelings of self-blame, guilt and anxiety about the way of cared for their babies. For some, this manifested as persistent worry about their child’s health. One mother reflected, ‘I felt like it was my fault, like I hadn’t done enough to take care of myself during pregnancy. I kept wondering if my baby would ever catch up’ (P07, 30 years, married). Another mother shared the emotional toll of her baby’s stay in the neonatal intensive care unit (NICU), saying, ‘My baby was in the NICU, and I couldn’t see him for 3 days. I kept thinking he wouldn’t survive, and it broke me’ (P08, 27 years, married). Physical changes following childbirth further compounded these struggles, with many mothers reporting diminished self-esteem and feelings of unattractiveness owing to postpartum weight gain and other bodily changes. One mother explained, ‘I look at my body now, and I don’t recognise myself. It makes me feel unattractive and unworthy’ (P10, 28 years, married). The caregiving challenges and low self-esteem, particularly among mothers of low-birthweight babies, contributed to heightened anxiety, self-blame and feelings of failure.

Moreover, some women compared their perceived shortcomings to the successes of their peers, which intensified feelings of inferiority. As one mother explained, ‘I see my friends doing so well, and I feel like I’m struggling just to get through the day. It makes me feel like I’ll never be good enough’ (P12, 39 years, cohabiting). These narratives underscore the impact of negative self-perception on mothers’ emotional well-being, increasing their vulnerability to postpartum depression.

#### Resilience resources

Many mothers exhibited resilience in the presence of postpartum struggles. Despite a lack of support, some mothers expressed resilience and determination to raise their babies. For instance, one mother stated, ‘Even though I am alone in this, I find joy in caring for my baby. Every moment I spend with her makes me feel complete. I may not have support, but I will not let that affect her well-being’ (P11, 27 years, not in union).

Furthermore, some mothers found moments of healing and emotional renewal through their connection with their babies. For some, the experience of caring for their child brought a sense of purpose and improved their mental well-being. One mother remarked, ‘After birth, I felt better. Even though the depression had set in, holding my baby and caring for him brought me a sense of peace and purpose. I felt I could do it, despite my history of depression’ (P12, 39 years, cohabiting). Similarly, others found solace in the support of family members and healthcare workers. This support included emotional encouragement from nurses during hospital visits and practical help from family (e.g., a relative caring for the infant or household duties), which mothers said gave them strength. A participant shared that, ‘The support from the nurses and my family helped me feel strong again. I began to find joy in motherhood despite my history’ (P13, 21 years, married).

Other mothers also expressed a sense of pride and self-worth as they came to appreciate their postpartum body. One mother remarked, ‘It took time, but I’m starting to appreciate my body for what it has done. I carried life, and that makes me proud’ (P14, 28 years, married). Encouragement from partners also played a role in fostering emotional resilience, as one mother shared, ‘My partner reminded me every day that I looked beautiful even with the stretch marks. It helped me see myself differently, not just for how I looked but for what my body had accomplished’ (P15, 31 years, cohabiting). Although some mothers remain deeply affected by social and emotional vulnerabilities, others find pathways to healing and resilience through meaningful connections and support systems.

### Integration of findings

We combined the quantitative and qualitative results. We found that factors that were significantly associated with PPDS (i.e. highest aPRs and *P* < 0.05) aligned with mothers’ narratives. For example, lack of partner support (aPR = 1.60) corresponded closely to the ’social isolation and limited support’ theme, and low self-esteem (aPR = 2.38) matched the ’self-image and identity challenges’ theme. Conversely, qualitative themes such as personal resilience added context beyond the survey. In this way, meta-integration showed that the main statistical correlates were echoed in lived experiences, reinforcing their importance.

## Discussion

### Main findings

Our findings showed a high PPDS prevalence of 29.3%, with key factors including being unmarried, lack of partner support, history of depressive or psychiatric disorders, unplanned pregnancy, low self-esteem and low-birth-weight infants. In-depth interviews further identified themes of social isolation, emotional stress, self-perception challenges and resilience. These offer insights into the mechanisms linking these factors to PPDS. Our results highlight the need for robust support systems and culturally sensitive interventions to prevent and reduce PPDS.

### Strengths and limitations

We combined quantitative analysis with rich qualitative insights, which provided a comprehensive understanding of these factors and mechanisms of PPDS. We used validated and practical psychometric instruments like the PHQ-9. However, the cross-sectional design limits determining causality. Also, our sample was predominantly urban (95%), reflecting the facility locations in Ho. This urban bias may limit generalisability to rural Ghana; rural mothers often face additional stressors and access barriers. Future research should also include rural communities to capture potential differences. Additionally, social desirability bias may have influenced responses on sensitive topics. Although the PHQ-9 has shown acceptable psychometric properties in Ghana (Cronbach’s alpha = 0.75),^21^ not all instruments (e.g. CEQ2, Rosenberg Self-Esteem Scale) were validated in Ewe. Translation issues arising from a lack of cultural adaptation may have introduced measurement error, which we cannot completely rule out. Again, our sample excludes mothers who do not attend postnatal clinics (for example, those giving birth at home or dropping out of care). This may bias results if such women have different PPDS rates.

### Interpretation

The prevalence of PPDS in our study is higher than the African average (22.1%) and previous estimates in Ghana (20.3%),^1,2^ suggesting potential contextual factors that increase vulnerability. Regional differences in Ghana showed lower PPDS in the Northern region (16.8%)^26^ and higher PPDS in the Ashanti region (31.4%),^27^ influenced by variations in healthcare systems, cultural norms and support structures.^28^ For example, extended family support in rural areas like the Northern region may offer protection against PPDS,^29^ whereas urbanisation in the Ashanti region, despite its development, contributes to reduced family support, increased work–family pressures and social isolation.^30^ In Ghana’s urbanised context, nuclear family living and changing gender roles may reduce traditional family support for new mothers. Additionally, in migration studies, Volta migrants depend heavily on family ties, whereas Northern migrants rely more on friends,^31^ reflecting distinct social networks. These socio-structural differences (including Volta’s proximity to Accra versus Northern Ghana’s remoteness) may affect support for mothers and thus help explain the regional prevalence patterns of PPDS that were observed. These findings underscore the importance of tailoring PPDS interventions to specific regional contexts.

Our findings suggest that PPDS among mothers in Ghana is influenced by a combination of sociodemographic, psychological, maternal and perinatal factors, with qualitative analyses highlighting pathways through which these factors exert their influence. Consistent with previous studies, key factors associated with PPDS include being unmarried, lack of partner support, history of depression or psychiatric disorders, unplanned pregnancy,^5^ low self-esteem^6^ and low birth weight.^7^ Being unmarried and experiencing unplanned pregnancies heightened feelings of societal isolation and inadequacy, reflecting cultural norms that stigmatise unmarried mothers.^32^

Our results align with Ghana’s patriarchal family norms, which emphasise that women should marry and bear children.^33^ In this context, an adolescent or unmarried pregnancy is widely viewed as shameful. Indeed, local qualitative studies report that unmarried pregnant adolescents are often verbally abused or ostracised by family and neighbours.^32,34^ The absence of partner support left mothers feeling isolated, emotionally strained and financially burdened, reinforcing the protective role of partner involvement in reducing emotional distress and improving maternal mental health.^35^

Qualitatively, we also observed that low self-esteem and caregiving challenges, particularly among mothers of low-birth-weight babies, contributed to heightened anxiety, self-blame and feelings of failure. This is consistent with a previous study highlighting the psychological distress caused by added caregiving demands^7^ and body image concerns. Societal expectations of motherhood further contributed to negative self-image and inadequacy.^36^ Many mothers exhibited resilience in the presence of PPDS. Some mothers found strength through body acceptance^11^ and displayed resilience as a result of emotional connection with their babies and support from family and healthcare workers.^37^ Other possible factors that promote resilience may include individual coping strategies, social support networks and cultural or spiritual beliefs. In our interviews, mothers who reported higher resilience often mentioned faith, encouragement from family or finding purpose in motherhood. Altogether, these findings underscore the dual nature of postpartum experiences, balancing adversity with opportunities for emotional growth. They also emphasise the need for culturally sensitive interventions, combining structural support, such as antenatal and postnatal classes, with psychological care to enhance resilience and reduce the burden of PPDS.^38^

Our findings have implications for Ghana’s health sector, informing public health policy, clinical practice and future research. At the policy level, it is essential to integrate maternal mental health services into existing maternal and child health programmes. This aligns with Ghana’s mental health policy, which advocates mental health integration into primary care. To leverage identified resilience factors, we recommend couple-based antenatal education (to involve partners in pregnancy care) and mother-to-mother support networks. Specifically, implementing partner-inclusive counselling and creating peer-mentor schemes could harness family and community support.^39^ Evidence shows that peer-support groups improve maternal mental health outcomes.^40^ Embedding such programmes within existing maternal health services could enhance the social support infrastructure for at-risk mothers.^38^ Clinically, maternal health service providers should implement routine screening for postpartum depression at postnatal visits and offer psychosocial support or referrals for mothers at high risk, such as those with prior psychiatric history or low social support. Future research should include longitudinal studies to validate these risk factors over time and the design of targeted interventions (such as partner-inclusive counselling or resilience-building programmes) to determine effective strategies for reducing the burden of postpartum depression in diverse Ghanaian settings.

In conclusion, PPDS were common in our sample and were significantly associated with modifiable factors such as prior mental health history and low self-esteem. These findings underscore the importance of prioritising maternal mental health through integration of depression screening and psychosocial care into routine antenatal and postnatal services, with particular support for high-risk groups like unmarried mothers.

## Supporting information

Tornyevah et al. supplementary material 1 Tornyevah et al. supplementary material

Tornyevah et al. supplementary material 2 Tornyevah et al. supplementary material

Tornyevah et al. supplementary material 3 Tornyevah et al. supplementary material

Tornyevah et al. supplementary material 4 Tornyevah et al. supplementary material

Tornyevah et al. supplementary material 5 Tornyevah et al. supplementary material

## Data Availability

Data will be made available to any interested researchers upon request. To request data access, one must write to the corresponding author, mentioning the intended use for the data. Data-set request must include contact information, a research project title and a description of the analysis being proposed, as well as the format it is expected in. The requested data should only be used for the purposes related to the original research or study.

## Supplementary material

The supplementary material is available online at https://doi.org/10.1192/bjo.2025.10857
