# Supplementary material for: Prevalence and factors associated with postpartum depressive symptoms among mothers who gave birth within the past 12 months in Ghana: mixed-method study
Source: BJPsych Open. 2025 Oct 14;11(6):e239. doi: 10.1192/bjo.2025.10857 (PMC12529339; doi:10.1192/bjo.2025.10857)
Supplement: Tornyevah et al. supplementary material 1 — Tornyevah et al. supplementary material [file S2056472425108570sup001.docx]

**QUALITATIVE INTERVIEW GUIDE**

**Thank you for agreeing to speak with us. We are interested in hearing about your experiences after childbirth. There are no right or wrong answers, so please share as much or as little as you feel comfortable. Everything you say will be kept confidential, and you can stop at any time.**

1. Could you please share with me your experiences since having your baby?
2. Could you please describe a typical day for you since your baby was born?
3. Can you share any significant experiences you remember from your pregnancy, delivery, or early time with your baby?

Prompt: “Could you please tell me more about what happened or how it felt?”

1. Could you describe the support or assistance you’ve received since your baby was born?
2. How have relationships with your partner, family, friends, or others affected your experiences?
3. Are there times when you were on your own? How did that feel?
4. Could you please tell me about how you have been feeling since giving birth and whether you’ve noticed any changes in your emotions or mood during this time?

Prompt: “Have there been days when you felt different emotions, like happier or more stressed than usual?”

1. How have responsibilities for your baby affected your daily life or feelings, if at all?
2. How has your day-to-day life changed since the baby was born?
3. How do you feel about yourself or your body since giving birth?
4. Could you please describe any ways that interactions with family, friends, or your community have influenced your experience as a mother?
5. Do you notice any comparisons between yourself and other mothers? How does that affect you, if at all?
6. Share with me what strategies you use to manage the challenges of caring for your baby.

Prompt: “Could you please give an example of a strategy, person, or resource that helps?”

1. Could you please share any experiences, strategies, or sources of support that have helped you feel better or more capable after childbirth?
