# Supplementary material for: Prevalence and factors associated with postpartum depressive symptoms among mothers who gave birth within the past 12 months in Ghana: mixed-method study
Source: BJPsych Open. 2025 Oct 14;11(6):e239. doi: 10.1192/bjo.2025.10857 (PMC12529339; doi:10.1192/bjo.2025.10857)
Supplement: Tornyevah et al. supplementary material 2 — Tornyevah et al. supplementary material [file S2056472425108570sup002.docx]

# Supplementary Table S2: Coding Tree : Themes, Subthemes, Codes, and Illustrative Quotes from Mothers Experiencing Postpartum Depressive Symptoms

| **THEME** | **SUBTHEMES** | **CODES** | **ILLUSTRATIVE QUOTES** |
| --- | --- | --- | --- |
| ****Social isolation and limited support**** | Lack of partner support | Absence during childbirth | “He wasn’t there when I needed him most, especially during labour and those first few weeks after birth.” **(P01, 34 years, married)** |
|  |  | Lack of emotional support | “I needed someone to talk to, someone to understand how I feel, but my partner just wasn’t there emotionally.” **(P02, 32 years, cohabiting)** |
|  |  | Insufficient financial support | “My partner barely supports us financially. I feel overwhelmed and alone.” **(P03, 42 years, married)** |
|  | Societal stigma and family rejection | Stigma for being unmarried | “Society expects us to marry first, then have children. Because I didn’t, people insult me. It hurts me every day.” **(P18, 22 years, not in union)** |
|  |  |  | *“Society expects too much from mothers. My own is even worse because I am not married”* ***(P11, 27 years, not in union)*** |
|  |  | Family disappointment | “My family said I disappointed them because I got pregnant before finishing school. They made me feel like a failure.” **(P19, 20 years, not in union)** |
|  |  | Social exclusion from peers | “When I got pregnant at 19, my friends started avoiding me. They insulted me, and I felt ashamed.” **(P05, 19 years, cohabiting)** |
| ****Emotional distress and vulnerability**** | Preexisting mental health conditions | Depression resurfacing | “I’ve had depression before, but this time it feels worse because my family has rejected me, and I’m doing everything alone.” ***(P05, 19 years, cohabiting)*** |
|  |  |  | *“I’ve had depression before, and it came back stronger after delivery.”* **(P17, 31 years, married)** |
|  | Caregiving stress | Worsening symptoms after childbirth | “I was already struggling mentally before I got pregnant. After the baby came, it became worse because I had no one to talk to.” **(P06, 31 years, separated)** |
|  |  |  | *“I always had anxiety during pregnancy, and after birth, it spiked because I felt so overwhelmed.”* **(P13, 21 years, married)** |
| ****Self-image and identity challenges**** | Self-blame and guilt | Feeling responsible for baby’s health | “I felt like it was my fault, like I hadn’t done enough to take care of myself during pregnancy. I kept wondering if my baby would ever catch up.” **(P07, 30 years, married)** |
|  |  |  | *“Seeing my baby so tiny made me question if I had done something wrong during pregnancy****.”* (P06, 31 years, separated)** |
|  |  | NICU-related anxiety | “My baby was in the NICU, and I couldn’t see him for three days. I kept thinking he wouldn’t survive, and it broke me.” **(P08, 27 years, married)** |
|  |  |  | *“At the hospital, I couldn’t even hold my baby for the first few days. She was at the NICU because of her low weight. It felt like I was failing as a mother.”* **(P01, 34 years, married)** |
|  | Physical changes | Body dissatisfaction | “I look at my body now, and I don’t recognize myself. It makes me feel unattractive and unworthy.” **(P10, 28 years, married)** |
|  |  |  | “*The weight gain, the scars, the changes in my body, I just feel unattractive. It’s hard to feel good about myself.”* **(P06, 31 years, separated)** |
|  |  |  | *“After giving birth, I felt like I didn’t look like myself anymore. It was hard to even look in the mirror.”* **(P13, 21 years, married)** |
|  | Comparison with peers | Inferiority compared to peers | “I see my friends doing so well, and I feel like I’m struggling just to get through the day. It makes me feel like I’ll never be good enough.” **(P12, 39 years, cohabiting)** |
|  |  |  | *“When I see my friends going to school I feel very sad. I am here struggling to take care of a baby”* **(P16, 23 years, cohabiting )** |
| ****Resilience resources**** | Inner strength and determination | Joy in caring for baby despite lack of support | “Even though I am alone in this, I find joy in caring for my baby. Every moment I spend with her makes me feel complete. I may not have support, but I will not let that affect her well-being.” **(P11, 27 years, not in union)** |
|  | Emotional healing through connection with baby | Bonding with baby brings peace and purpose | “After birth, I felt better. Even though the depression had set in, holding my baby and caring for him brought me a sense of peace and purpose. I felt I could do it, despite my history of depression.” **(P12, 39 years, cohabiting)** |
|  | Support systems as resilience enablers | Emotional encouragement from healthcare workers | “The support from the nurses and my family helped me feel strong again. I began to find joy in motherhood despite my history.” **(P13, 21 years, married)** |
|  |  | Practical help from family | “The help I got from relatives gave me strength.” **(P19, 22 years, not in union)** |
|  | Positive self-image and body appreciation | Pride in postpartum body | “It took time, but I’m starting to appreciate my body for what it has done. I carried life, and that makes me proud.” **(P14, 28 years, married)** |
|  |  | Partner encouragement fostering self-worth | “My partner reminded me every day that I looked beautiful even with the stretch marks. It helped me see myself differently, not just for how I looked but for what my body had accomplished.” **(P15, 31 years, cohabiting)** |
