# Supplementary material for: Prevalence and factors associated with postpartum depressive symptoms among mothers who gave birth within the past 12 months in Ghana: mixed-method study
Source: BJPsych Open. 2025 Oct 14;11(6):e239. doi: 10.1192/bjo.2025.10857 (PMC12529339; doi:10.1192/bjo.2025.10857)
Supplement: Tornyevah et al. supplementary material 5 — Tornyevah et al. supplementary material [file S2056472425108570sup005.docx]

**Supplementary Table S4: Background Charateristics Of Interview Participants**

| Participant ID | Age (yrs) | Level of Education | Employment Status | Marital Status | Religion | Parity | Mode of Delivery | Time Since Delivery |
| --- | --- | --- | --- | --- | --- | --- | --- | --- |
| P01 | 34 | Tertiary | Self-employed | Married | Christian | 2 | C.S | 6 months |
| P02 | 32 | Upper Secondary (SHS) | Self-employed | Cohabiting | Christian | 2 | C.S | 2 months |
| P03 | 42 | Upper Secondary(SHS) | Self-employed | Married | Christian | 4 | C.S | 2 months |
| P04 | 28 | Tertiary | Unemployed | Cohabiting | Christian | 1 | C.S | 10 months |
| P05 | 19 | Upper Secondary(SHS) | Unemployed | Cohabiting | Christian | 1 | C.S | 8 months |
| P06 | 31 | Lower Secondary(JHS) | Self- employed | Separated | Christian | 4 | C.S | 3 months |
| P07 | 30 | Upper Secondary(SHS) | Unemployed | Married | Christian | 3 | C.S | 3 months |
| P08 | 27 | Tertiary | Employed | Married | Muslim | 1 | C.S | 2 months |
| P09 | 27 | Tertiary | Employed | Cohabiting | Christian | 2 | C.S | 2 months |
| P10 | 28 | Primary | Self-employed | Married | Christian | 3 | C.S | 2 months |
| P11 | 27 | Upper Secondary(SHS) | Unemployed | Not in union | Christian | 1 | C.S | 5 months |
| P12 | 39 | Upper Secondary(SHS) | Self-employed | Cohabiting | Christian | 3 | C.S | 3 months |
| P13 | 21 | No education | Self-employed | Married | Christian | 2 | SVD | 2 months |
| P14 | 28 | Tertiary | Employed | Married | Muslim | 2 | SVD | 7 months |
| P15 | 31 | Upper Secondary(SHS) | Self-employed | Cohabiting | Christian | 3 | SVD | 9 months |
| P16 | 23 | Lower Secondary(JHS) | Unemployed | Cohabiting | Muslim | 2 | SVD | 2 months |
| P17 | 31 | Tertiary | Employed | Married | Muslim | 1 | SVD | 2 months |
| P18 | 22 | Lower Secondary(SHS) | Self-employed | Not in union | Muslim | 2 | SVD | 4 months |
| P19 | 20 | Lower Secondary(JHS) | Self-employed | Not in union | Muslim | 1 | SVD | 6 months |

JHS=Junior Secondary school, SHS=Senior Secondary School, C.S= Caesarean Section, SVD= Spontaneous Vagina Delivery
